# Dietary Intake of 17α-Ethinylestradiol Promotes HCC Progression in Humanized Male Mice Expressing Sex Hormone-Binding Globulin

**DOI:** 10.3390/ijms222212557

**Published:** 2021-11-22

**Authors:** Sang R. Lee, Su Hee Jeong, Jun H. Heo, Seong Lae Jo, Je-Won Ko, Hyo-Jung Kwun, Eui-Ju Hong

**Affiliations:** College of Veterinary Medicine, Chungnam National University, Daejeon 34134, Korea; srlee5@cnu.ac.kr (S.R.L.); 719shjung@naver.com (S.H.J.); heojh0624@naver.com (J.H.H.); jsr7093@o.cnu.ac.kr (S.L.J.); rheoda@cnu.ac.kr (J.-W.K.); hyojung@cnu.ac.kr (H.-J.K.)

**Keywords:** 17α-ethinylestradiol, SHBG, HCC, liver cancer, EE2, androgen

## Abstract

Hepatocellular carcinoma (HCC) is a male-oriented malignancy; its progression is affected by sex hormones. 17α-ethinylestradiol (EE2) is a synthetic estrogen widely used as an oral contraceptive; however, it is unknown whether EE2 regulates sex hormone action in HCC. We investigated whether EE2 influences HCC risk in male androgenic environments, using mice expressing human sex hormone-binding globulin (SHBG). Two-week-old male mice were injected with diethyl-nitrosamine (DEN, 25 mg/kg) and fed an EE2 diet for 10 weeks from 30 weeks of age. Development and characteristics of liver cancer were evaluated in 40-week-old mice via molecular and histological analyses. Although EE2 did not increase HCC progression in wild-type mice, SHBG mice exhibited remarkably higher HCC risk when fed EE2. The livers of EE2-treated SHBG mice exhibited substantially increased pro-inflammatory necrosis with high plasma levels of ALT and HMGB1, and intrahepatic injury and fibers. Additionally, increased androgen response and androgen-mediated proliferation in the livers of EE2-treated SHBG mice and EE2-exposed hepatocytes under SHBG conditions were observed. As a competitor of SHBG-androgen binding, EE2 could bind with SHBG and increase the bioavailability of androgen. Our results revealed that EE2 is a novel risk factor in androgen-dominant men, predisposing them to HCC risk.

## 1. Introduction

As the female hormone 17β-estradiol (E2) is a potent suppressor of the hepatic pro-inflammatory response, which is essential for liver cancer progression, hepatocellular carcinoma (HCC) is considered a female-protective neoplasm [1]. In contrast, because androgen and androgen receptors are both considered HCC-promoters [2], men are at a much higher risk of developing HCC than women because of their high androgen and low estrogen levels [3].

Sex hormone-binding globulin (SHBG), a sex steroid hormone regulator, binds to sex steroid hormones, such as androgen and estrogen. It is well known that sex steroid hormones bound to SHBG lose their bioavailability; however, it has also been suggested that SHBG enhances the ability of sex steroid hormones to target tissues [4]. SHBG not only circulates in the bloodstream, but also accumulates in specific compartments, which intensifies their steroid-regulatory action [5]. It has been reported that androgen regulation by SHBG occurs in male reproductive organs and androgen-responsive organs, such as the prostate [6] and kidney [7]. Nonetheless, it is difficult to determine whether SHBG increases or decreases androgenic action due to organ differences in its cellular location. Additionally, although SHBG in the female reproductive organs suppresses estrogenic action [6], the post-menopausal estrogenic was accentuated by SHBG in a high-fat-fed liver [8], suggesting that hormonal regulation by SHBG is also affected by physiological sex hormone concentrations. This indicates that the regulation of sex steroid hormones by SHBG is not coherent under all physiological conditions; nevertheless, SHBG can be regarded as a crucial sex steroid hormone modulator and could be involved in sex steroid hormone-related pathogenesis.

17α-ethinylestradiol (EE2), a synthetic estrogen, has long been considered a favorable component of contraceptives and constitutes the first-line therapy for contraception in women [9]. However, it was observed that a constant dietary intake of EE2 promotes hepatocarcinogenesis in rats [10,11]. Furthermore, a previous study reported that long-term administration of EE2 leads to hepatic neoplasm [12]. Nonetheless, a recent large analysis showed that women taking oral contraceptives are not vulnerable to HCC development [13]. Meanwhile, EE2 has been used in men as a prostate cancer therapy for several decades [14], because it shows high clinical efficacy in castration-resistant prostate cancer patients [15,16]. Although it was reported prostate cancer patient can be exposed to liver cancer following estrogen therapy [17], the side effects for EE2 intake in men are not well known, particularly regarding HCC risk. In this study, we compared the development of HCC in mice fed a regular diet with that in mice fed an EE2-containing diet. At 40 weeks of age, EE2 did not promote HCC progression in wild-type (WT) mice. However, mice expressing human SHBG were substantially more prone to HCC progression when fed the EE2-containing diet; this resulted in increased necrosis and proliferation of liver cells. Considering that EE2 has an affinity for SHBG, which is a crucial steroid hormone regulator, a possible mechanism of EE2 action in the presence of SHBG was investigated in HCC-bearing livers of mice.

## 2. Results

### 2.1. EE2 Promotes HCC Progression in SHBG Mice

**Figure 1:**
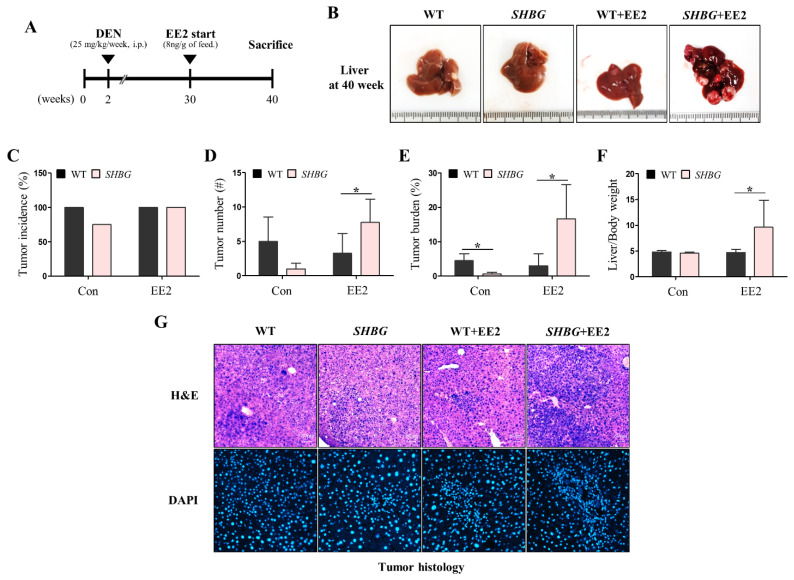
HCC development of EE2-fed WT and *SHBG* mice. (**A**) Experimental schedule for inducing HCC and feeding EE2. DEN (25 mg/kg, IP) was injected to 2-week-old mice. EE2 was dissolved in diet and fed to mice from the age of 30-weeks-old. Mice were sacrificed at 40-weeks of age. (**B**) Representative liver images of WT, *SHBG*, WT EE2, *SHBG* EE2 mice. (**C**) Tumor incidence of WT, *SHBG*, WT EE2, *SHBG* EE2 mice. (**D**) Tumor number of WT, *SHBG*, WT EE2, *SHBG* EE2 mice. Each tumor numbers were counted per liver. (**E**) Tumor burden of WT, *SHBG*, WT EE2, *SHBG* EE2 mice. Each tumor burden per liver was calculated from ratio of the tumor area per total liver area. Image J software was used for an analysis. (**F**) Liver per body weight of WT, SHBG, WT EE2, SHBG EE2 mice. (**G**) Hematoxylin & Eosin (H&E) and DAPI staining in tumor-bearing livers of WT, SHBG, WT EE2, SHBG EE2 mice. Scale bar; 80 µm. Numbers of mice used for experiments were: WT (4), *SHBG* (4), WT EE2 (8), *SHBG* EE2 (8). Student’s *t*-Test was used for analysis. Values represent means ± SD. * *p* < 0.05.

Mice were injected with DEN and fed a normal diet or an EE2-containing diet, as shown in Figure 1A. Representative gross images of 40-week-old livers are presented in Figure 1B. WT livers possessed observable tumor foci, whereas *SHBG* livers presented very small tumor nodules. Although tumor foci were similar in WT EE2 livers and WT livers, they were substantially larger in *SHBG* EE2 livers than in *SHBG* livers. Tumor incidence was 75% in SHBG livers, but WT, WT EE2, and *SHBG* EE2 livers exhibited 100% carcinogenesis (Figure 1C).

*SHBG* livers had lower tumor numbers compared with WT livers, although the difference was not significant, likely because of the small number of mice used for the experiment. WT EE2 livers did not show an increased number of tumors compared to WT livers; however, SHBG EE2 livers presented a greater number of tumor nodules than SHBG livers. Furthermore, SHBG EE2 livers possessed a higher number of tumors than the WT EE2 livers (Figure 1D).

When individual tumor burden was estimated and calculated for each liver, *SHBG* livers had significantly reduced tumor size compared with WT livers. Although WT EE2 livers did not exhibit an increased tumor burden compared to WT livers, *SHBG* EE2 livers had larger tumors than *SHBG* livers had. Furthermore, SHBG EE2 livers contained substantially larger tumors than those in WT EE2 livers (Figure 1E). Body weights were lower in EE2-fed groups, but there was no difference between body weights of WT EE2 and *SHBG* EE2 mice (Figure S1A). Liver weight and liver per body weight were both significantly increased in *SHBG* EE2 mice compared with WT EE2 mice, which may be induced by tumor weight (Figure S1B,F). In H&E staining, tumors exhibited similar histopathological characteristics presenting aggregation of condensed nuclei (Figure 1G). Particularly, SHBG EE2 tumor showed a larger area of aggregated nuclei than other tumors (Figure 1G). These results indicate that EE2-induced HCC progression requires sex hormone regulation by SHBG in mice, suggesting that EE2 effects are accentuated or EE2 affects other sex hormone actions of SHBG.

### 2.2. EE2 Exacerbates Hepatic Necrosis in SHBG Mice

**Figure 2:**
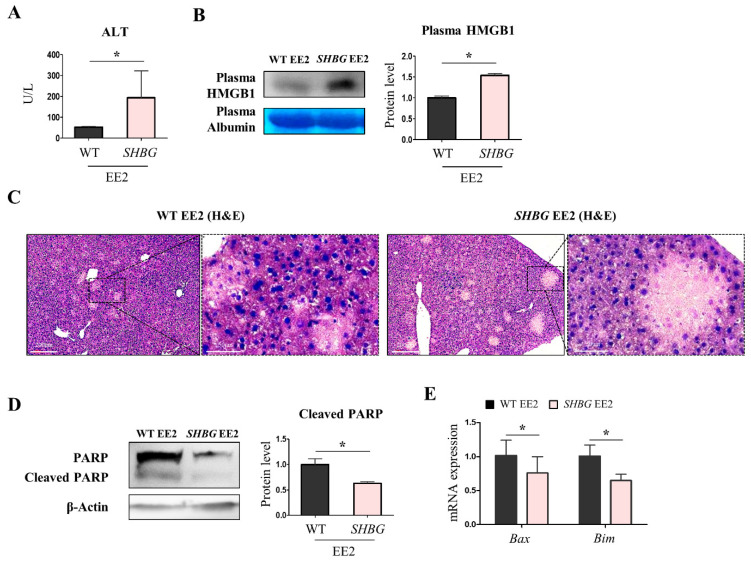
Hepatic necrosis was increased in *SHBG* mice fed with EE2. (**A**) Plasma ALT level (U/L) of WT EE2 and *SHBG* EE2 mice. (**B**) Western blot analysis and quantification of plasma HMGB1 in WT EE2 and *SHBG* EE2 mice. Plasma albumin was used for an internal control. (**C**) H&E staining in livers of WT EE2 and *SHBG* EE2 mice. Scale bar; 200 µm and 50 µm. (**D**) Western blot analysis and quantification of intact and cleaved PARP in livers of WT EE2 and *SHBG* EE2 mice. β-Actin was used for an internal control. (**E**) mRNA expression of *Bax* and *Bim* in livers of WT EE2 and *SHBG* EE2 mice. *Rplp0* was used for an internal control. Numbers of mice used for experiments were: WT EE2 (8), *SHBG* EE2 (8). Student’s *t*-Test was used for analysis. Values represent means ± SD. * *p* < 0.05. Data were quantified from replicated values in which independent experiments were performed in triplicate at least.

Next, we investigated whether EE2 affects hepatic injury by estimating physiological and molecular markers in the plasma and livers of mice. Necrosis is a pro-inflammatory hepatic injury that is closely associated with the risk of HCC [18]. As a hepatic necrosis marker, the level of alanine aminotransferase (ALT) was markedly increased in SHBG EE2 plasma compared with that in WT EE2 plasma (Figure 2A). Additionally, as a hepatic necrosis marker, the protein levels of plasma high mobility group box 1 (HMGB1) were increased in SHBG EE2 plasma (Figure 2B). H&E staining results showed pronounced hepatic necrosis in SHBG EE2 livers, which had massive pale areas without the presence of hepatocytes (Figure 2C).

Contrary to necrosis, apoptosis rarely triggers a pro-inflammatory response because apoptotic vesicles are formed rather than being disseminated and this might suppress the inflammatory response [19]. Hence, apoptosis can dispose of abnormal cells in an adequate manner, which barely induces proliferation. Western blotting results revealed that the protein levels of cleaved PARP were suppressed in SHBG EE2 livers compared with those in WT EE2 livers (Figure 2D). Furthermore, the mRNA expression levels of Bcl2 associated X (*Bax*) and Bcl-2-like 11 (*Bim*) were reduced in SHBG EE2 livers (Figure 2E). In contrast to hepatic injury and necrosis, the apoptotic response by EE2 was substantially suppressed with SHBG presence.

### 2.3. EE2 Increases Hepatic Fibrosis in SHBG Mice

**Figure 3:**
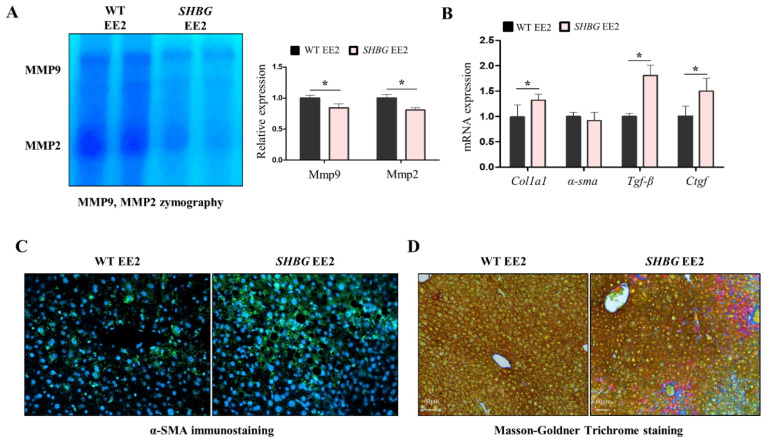
Hepatic fibrosis was induced in *SHBG* mice fed with EE2. (**A**) Zymographic analysis and quantification of MMP9 and MMP2 in livers of WT EE2 and *SHBG* EE2 mice. (**B**) mRNA expression of *Col1a1, α-Sma*, *Tgf-β*, and *Ctgf* in livers of WT EE2 and *SHBG* EE2 mice. *Rplp0* was used for an internal control. (**C**) Immunostaining of α-SMA (green) in livers of WT EE2 and *SHBG* EE2 mice. DAPI staining was used for an internal nucleus control. (**D**) Masson-Goldner Trichrome staining in livers of WT EE2 and *SHBG* EE2 mice. Fibers were stained with bright red. Scale bar; 60 µm. Numbers of mice used for experiments were: WT EE2 (8), *SHBG* EE2 (8). Student’s *t*-Test was used for analysis. Values represent means ± SD. * *p* < 0.05. Data were quantified from replicated values in which independent experiments were performed in triplicate at least.

Necrosis triggers pro-inflammatory responses because cell debris stimulates immune cell activation. Following unresolved inflammation, hepatic fibrosis can be induced [20]. Zymographic analysis showed low levels of matrix metalloproteinase-9 (Mmp-9) and matrix metalloproteinase-2 (Mmp-2) in SHBG EE2 livers (Figure 3A). The mRNA expression of collagen type I alpha 1 chain (*Col1a1*), trans-forming growth factor-beta (*Tgf-β*), and connective tissue growth factor (*Ctgf*) was significantly increased in SHBG EE2 livers (Figure 3B). Additionally, α-SMA immunofluorescence positive signals were more pronounced in SHBG EE2 livers (Figure 3C). Upon Masson-Goldner trichrome staining, bright red areas were more pronounced in SHBG EE2 livers, suggesting that intrahepatic fibers were accumulated (Figure 3D). These results suggest that SHBG EE2 livers are prone to fibrosis, which may be triggered by increased hepatic inflammation.

### 2.4. EE2 Increases Hepatic Pro-Inflammatory and Proliferation Responses in SHBG Mice

**Figure 4:**
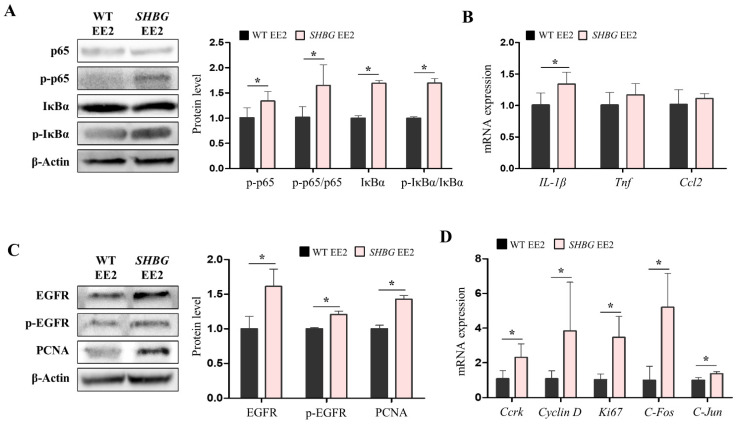
Pro-inflammatory response and compensatory proliferation were promoted in *SHBG* mice fed with EE2. (**A**) Western blot analysis and quantification of intact and phosphorylated p65 NF-κB and IκBα in livers of WT EE2 and *SHBG* EE2 mice. β-Actin was used for an internal control. (**B**) mRNA expression of *IL-1β*, *Tnf, Ccl2* in livers of WT EE2 and *SHBG* EE2 mice. *Rplp0* was used for an internal control. (**C**) Western blot analysis and quantification of intact and phosphorylated EGFR, and PCNA in livers of WT EE2 and *SHBG* EE2 mice. β-Actin was used for an internal control. (**D**) mRNA expression of *Ccrk*, *Cyclin D*, *Ki67*, *C-Fos*, and *C-Jun* in livers of WT EE2 and *SHBG* EE2 mice. *Rplp0* was used for an internal control. Numbers of mice used for experiments were: WT EE2 (8), *SHBG* EE2 (8). Student’s *t*-Test was used for analysis. Values represent means ± SD. * *p* < 0.05. Data were quantified from replicated values in which independent experiments were performed in triplicate at least.

Pro-inflammatory response was evaluated in livers by estimating levels of molecular markers. Western blot analysis revealed that the protein levels of p-nuclear factor kappa-light-chain-enhancer of activated B cells (NF-κB [p65]) and the ratio of p-p65 to p65 were both increased in SHBG EE2 livers compared with those in WT EE2 livers (Figure 4A). Likewise, the protein levels of p-NF-kappa-B inhibitor alpha (IκBα) and the ratio of p-IκBα to IκBα were increased in SHBG EE2 livers (Figure 4A). The mRNA expression of interleukin 1 beta (*IL-1β*) was significantly increased in SHBG EE2 livers (Figure 4B).

As pro-inflammatory responses are closely related to a compensatory proliferation response, which may be mediated by growth factors [21], we investigated the epidermal growth factor receptor (EGFR) activation. Results showed that the protein levels of EGFR and p-EGFR were both increased in SHBG EE2 livers compared with those in WT EE2 livers. Proliferating cell nuclear antigen (PCNA) protein levels were also increased in SHBG EE2 livers (Figure 4C). The mRNA expression of cell cycle-related kinase (*Ccrk*), *cyclin D*, *Ki67*, *C-Fos*, and *C-Jun* was significantly increased in SHBG EE2 livers (Figure 4D). These findings indicate that EE2 increases the proliferation response in SHBG livers, which may be triggered by enhanced pro-inflammatory responses.

### 2.5. The Presence of Plasma SHBG Accentuates Androgenic Effects

**Figure 5:**
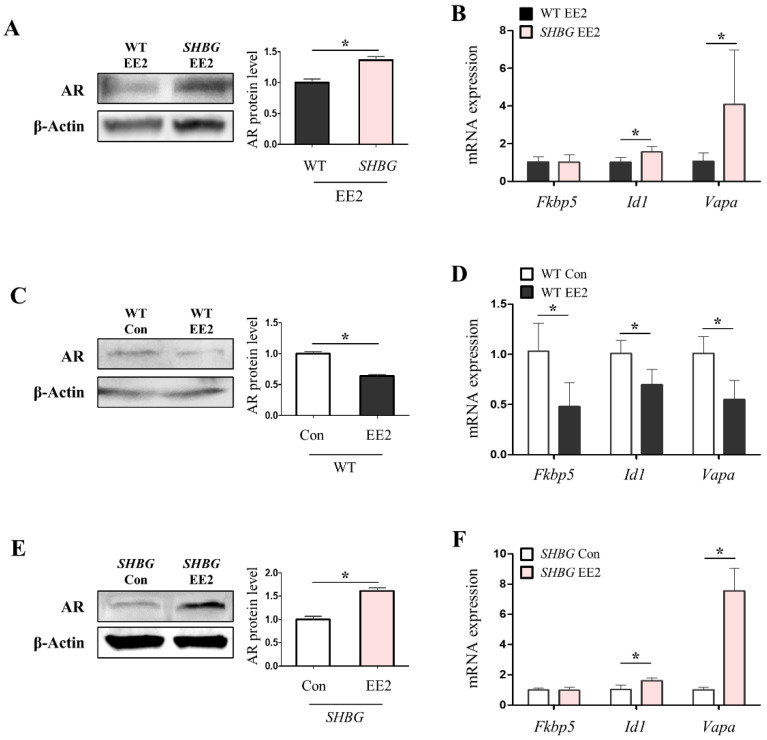
Androgenic effects were accentuated in *SHBG* mice fed with EE2. (**A**) Western blot analysis and quantification of AR in livers of WT EE2 and *SHBG* EE2 mice. β-Actin was used for an internal control. (**B**) mRNA expression of androgen-AR target genes, *Fkbp5*, *Id1*, and *Vapa*, in livers of WT EE2 and *SHBG* EE2 mice. *Rplp0* was used for an internal control. (**C**) Western blot analysis and quantification of AR in livers of WT and WT EE2 mice. β-Actin was used for an internal control. (**D**) mRNA expression of *Fkbp5*, *Id1*, and *Vapa* in livers of WT and WT EE2 mice. *Rplp0* was used for an internal control. (**E**) Western blot analysis and quantification of AR in livers of *SHBG* and *SHBG* EE2 mice. β-Actin was used for an internal control. (**F**) mRNA expression of *Fkbp5*, *Id1*, and *Vapa* in livers of *SHBG* and *SHBG* EE2 mice. *Rplp0* was used for an internal control. Numbers of mice used for experiments were: WT EE2 (8), *SHBG* EE2 (8). Student’s *t*-Test was used for analysis. Values represent means ± SD. * *p* < 0.05. Data were quantified from replicated values in which independent experiments were performed in triplicate at least.

The cancerous characteristics of SHBG EE2 livers were well addressed in this study. Additionally, the physiological conditions leading to the acceleration of necrosis and proliferation in SHBG EE2 livers were evaluated. As men are predisposed to cirrhosis [22], and the testosterone: estradiol ratio is associated with HCC risk in cirrhosis [23], androgenic effects were analyzed in EE2 livers. SHBG EE2 livers exhibited increased hepatic androgen receptor (AR) levels compared with WT EE2 livers, suggesting that androgen effects were accentuated (Figure 5A). This was further supported by the mRNA expression levels of AR target genes that were induced in SHBG EE2 livers (Figure 5B). When we investigated the effects of EE2 in the absence of SHBG, WT EE2 livers exhibited decreased AR levels and AR target gene levels compared with those in WT livers (Figure 5C,D). In contrast to WT livers, SHBG EE2 livers showed increased AR and AR target gene expression levels compared with SHBG livers that were not exposed to EE2 (Figure 5E,F). These results suggest that androgen effects could be accentuated in SHBG livers administered EE2, resulting in HCC progression by triggering deleterious conditions in SHBG EE2 livers.

**Figure 6:**
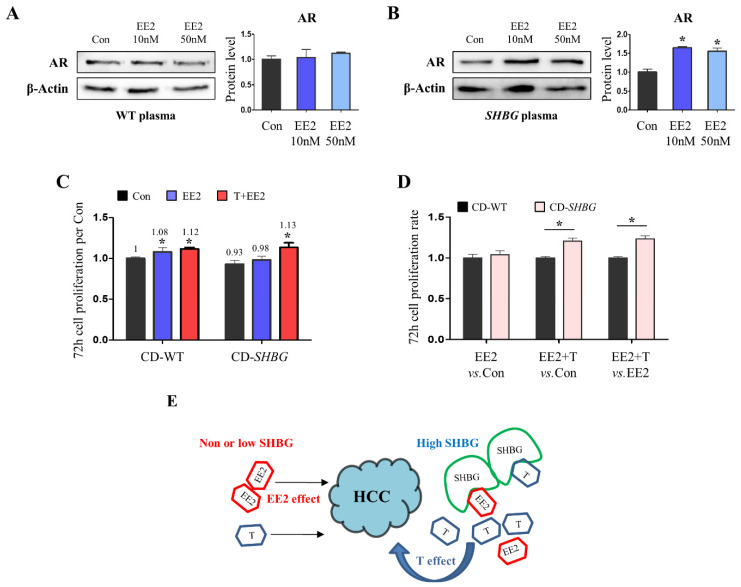
Androgenic effects were accentuated by EE2 in *SHBG* present SNU423 cells. (**A**) Western blot analysis and quantification of AR in vehicle or EE2-treated SNU423 cells with steroid-present WT plasma. β-Actin was used for an internal control. (**B**) Western blot analysis and quantification of AR in vehicle or EE2-treated SNU423 cells with steroid-present *SHBG* plasma. β-Actin was used for an internal control. (**C**) Cell proliferation rate of vehicle or EE2-treated SNU423 cells with CD-WT or CD-SHBG plasma after 72 h incubation. EE2 was treated for 48 h following 24 h of vehicle or testosterone (T) incubation. Cell proliferation rates were calibrated to 0 h incubation. Values were normalized to WT con. Steroid hormones were deprived by CD (charcoal dextran) incubation in WT and *SHBG* plasma. (**D**) Cell proliferation ratio as indicated; EE2 vs. Con, EE2 + T vs. Con, EE2 + T vs. EE2. (**E**) Schematic illustration of EE2-SHBG-T mechanism. Student’s *t*-Test and one-way ANOVA followed by Tukey’s post-hoc test were used for analysis. Values represent means ± SD. * *p* < 0.05. Data were quantified from replicated values in which independent experiments were performed in triplicate at least.

Next, we investigated whether SHBG potentiates androgenic action following EE2 treatment in HCC cells. To investigate androgenic effects, we used AR-overexpressing SNU423 cells. When SNU423 cells were exposed to EE2 with WT plasma, AR protein levels did not increase (Figure 6A). Conversely, SNU423 cells exhibited increased AR levels following EE2 treatment with SHBG plasma (Figure 6B), suggesting that SHBG plasma acts as an AR activator in EE2 treatment. Furthermore, CD-SHBG plasma (CD; charcoal dextran) increased cell proliferation following EE2 + T treatment, and the extent was greater than that obtained with CD-WT plasma (Figure 6C). As the induction of cell proliferation was more pronounced in the EE2 + T treatment than in the EE2 treatment (Figure 6D), testosterone likely acted as an activator of cell proliferation under the SHBG-EE2 + T condition. Based on the above-obtained in vivo and in vitro results, we described a possible mechanism regarding the interaction between EE2-SHBG-T (Figure 6E). In the absence of SHBG, EE2 treatment in males does not potentiate androgenic effects. However, in the presence of SHBG, EE2 treatment triggers the separation of testosterone from SHBG, thereby increasing the androgenic effect.

## 3. Discussion

EE2 is the most common component of oral contraceptives [24] because it possesses a substantially higher affinity for estrogen receptors than E2 [25]. In addition to preventing pregnancy, oral contraceptives are widely used among modern women to reduce the length of bleeding day during menstruation, although risks for oncogenic complications have yet to be investigated [26]. To observe the effect of EE2 in a humanized environment, mice expressing human *SHBG* were used in present study, considering that adult mice do not express *SHBG* in their blood [27]. EE2 was found to increase HCC risk only in humanized mice presenting plasma SHBG, whereas EE2 failed to promote HCC in the absence of SHBG. Although HCC progression was phenotypically suppressed in SHBG mice compared with that in WT mice, which is similar to our published data [28], EE2 could expose SHBG mice to HCC risk by triggering markedly pronounced hepatitis, fibrosis, and compensatory proliferation with AR activation. Although survival data are not included in the present study, it is expected that a substantially high number and burden of tumors might lead to a short survival period of HCC-bearing mice, similar to the previous study [29]. Our study highlights the clinical risk for men taking EE2, particularly those expressing high SHBG levels, and women ingesting EE2, particularly those with high androgen levels.

First, we characterized EE2-promoted HCC using molecular techniques. When external stimuli damaged liver cells, hepatocytes died via necrosis or apoptosis. Necrosis is cancerous cell death, which triggers immune cell activation, as disseminated cellular debris can be recognized by phagocytic receptors [30]. Necrosis is characterized by high plasma ALT and HMGB1 levels [31,32], which are increased in SHBG EE2 mice. Additionally, Mmp9 and Mmp2 mediate fibrosis resolution [33], which are suppressed in SHBG EE2 mice. *Col1a1*, *Tgf-β*, and *Ctgf* are chronic liver fibrotic markers [34,35,36], which are also induced in SHBG EE2 mice. Histological analysis via H&E, α-SMA immunostaining [37], and Masson-Goldner trichrome staining also revealed increased necrosis and fibrosis in SHBG EE2 livers. Conversely, apoptosis is programmed cell death, which is not closely related to a pro-inflammatory response. As apoptosis is a normal physiological phenome-non that reduces unusual cell accumulation upon injury [38], a decrease in cleaved PARP expression suggests that SHBG EE2 dampens the host-protective cell death machinery. Suppressed hepatic apoptosis is also supported by suppressed mRNA expression levels of apoptotic genes, including *Bax* and *Bim* [39]. Accordingly, our results suggest that SHBG EE2 triggers pro-oncogenic cell death in the liver, leading to necrosis and fibrosis.

Liver cancer is an inflammatory disease mainly caused by viral infection [40], and the constant activation of non-resolving inflammation leads to cancer cell proliferation [41]. Therefore, diverse therapeutic compounds targeting inflammation-induced chemokines, cytokines, and growth factors have been investigated in the past [42]. We analyzed whether HCC progression in SHBG EE2 mice is related to proliferation or inflammation-triggered proliferation. Results showed that SHBG EE2 substantially increased the growth factor response mediated by EGFR, which is known to be highly activated during hepatocarcinogenesis and is an inflammatory target for HCC therapy [29]. As a cell proliferation marker [43], PCNA levels were induced by SHBG EE2. Additionally, the higher expression of cell proliferation genes [43,44,45,46,47], *Ccrk*, *Cyclin D*, *Ki67*, *C-Fos*, and *C-Jun* indicated the induction of proliferation triggered by SHBG EE2. As a pro-inflammatory marker [48], p65 protein was highly phosphorylated in SHBG EE2, suggesting that a pro-inflammatory response could result in increased proliferation. This was also supported by the high mRNA expression levels of *Il-1β*¸ which is a macrophage activation marker [49]. In summary, the phenotype of SHBG EE2 triggered HCC progression via a pro-inflammatory response and increased proliferation.

Although the carcinogenic effect of EE2 was not observed in WT mice, it was well pronounced in SHBG mice. However, *SHBG* mice, who were not administered EE2, showed suppressed HCC progression compared with WT mice. SHBG is a sex steroid hormone-binding protein that regulates the bioavailability and access of sex steroid hormones [4]. The risk of EE2 intake in HCC bearing mice must be accentuated by SHBG. As the magnitude of the response of EE2 in terms of the actual increase in SHBG levels was much greater than the E2-mediated SHBG levels [50,51], a larger amount of SHBG (Figure S2) could amplify the carcinogenic effect of EE2. However, regarding HCC progression in WT mice with EE2 intake, the oncogenic effect of EE2 on HCC risk appeared negligible without SHBG.

Apart from the independent effect of EE2, the steroid hormone effect should also be considered. It is well known that androgens bound to SHBG are not bioavailable [52], but they can be specifically transported to their cytosolic receptor when SHBG approaches that compartment. As androgens have a markedly high affinity for SHBG compared with other sex steroids, their access to AR is highly limited by SHBG under normal physiological conditions. When EE2 binds to SHBG, the interaction between androgens and AR should be promoted because SHBG-unbound androgens in the blood are freely accessible to target tissues [4]. Considering the high activation of AR in SHBG mice and SHBG-present SNU423 cells, we concluded that EE2 accentuates androgenic effects by triggering androgen separation from SHBG, thereby increasing the levels of bioavailable androgen.

In summary, our study demonstrated that EE2 is particularly oncogenic when SHBG is primarily occupied by androgen, because EE2 increases bioavailable androgen. Although EE2 intake does not increase HCC susceptibility in women [13], it might be caused by the minimal plasma androgen levels throughout their reproductive years [53]. As endogenous or exogenous SHBG-binding chemicals can increase unbound androgen levels [54], EE2 intake in men expressing high SHBG will result in increase of unbound androgens and trigger HCC risk. Additionally, these findings should be taken into consideration while conducting clinical studies for EE2 usage in male patients with metastatic prostate cancer [55], as it can be particularly oncogenic.

## 4. Materials and Methods

### 4.1. Animals and Treatment

Male heterozygous human SHBG (4.3 kb)^+/−^ mice (*SHBG*-mice) [56] and WT mice on a C57BL/6N background were housed in a pathogen-free facility at Chungnam National University under a standard 12 h light: 12 h dark cycle and fed standard chow with water *ad libitum*. All animal experiments were approved and performed in accordance with the Chungnam Facility Animal Care Committee (CNU-00917). For HCC induction, 2-week-old mice were injected by diethylnitrosamine (DEN, 25 mg/kg, 73861; Sigma, Saint Louis, MO, USA) intraperitoneally. Mice were monitored by palpation for tumor formation and killed by cervical dislocation at the indicated time points for plasma and liver samples. Littermates were used for experiments in each of WT and SHBG-Tg mice without randomization. EE2 was dissolved in ethanol and injected to feeds (8 ng EE2 per 1 g of feed). Feeds were dried overnight to remove ethanol and fed to mice. Mice used for experiments were: 4 (WT), 4 (*SHBG*), 8 (WT EE2), 8 (*SHBG* EE2).

### 4.2. RNA Isolation, Reverse Transcription and qRT-PCR

cDNA was synthesized with 1 µg of total liver RNA and Excel RT Reverse transcriptase kit (SG-cDNAS100, Smartgene, Daejeon, Korea) following the manufacturer’s protocol. Quantitative RT-PCR was carried out using specific primers (Table 1), Excel Taq Q-PCR Master Mix (SG-SYBR-500, Smartgene), and Stratagene Mx3000P (Agilent Technologies, Santa Clara, CA, USA) equipped with a 96-well optical reaction plate. Negative controls containing water without sample cDNA were used in each plate. All experiments were performed in triplicate and mRNA values were calculated based on the cycle threshold after monitoring melting curve.

### 4.3. Western Blotting

Protein homogenates from liver and SNU423 cell were subjected to SDS-PAGE electrophoresis, and gels were transferred to the PVDF membrane (IPVH 00010, Millipore, Burlington, MA, USA). The membranes were blocked and incubated with primary antibodies (see below) in tube rotator for overnight at 4 °C. Membranes were 3-times-washed in TBS-T to remove non-specific antibody and then incubated with secondary antibodies (Goat anti-Rabbit IgG HRP; Catalog # 31460, Goat anti-Mouse IgG HRP; Catalog # 31430, Thermo Fisher Scientific, Waltham, MA, USA) for overnight at 4 °C. Following 3 washes in TBS-T, immunoreactive proteins were detected using Ultra 2.0 Western Blotting substrate (XLS075, 1000, Cyanagen, Bologna, Italia) ECL solution.

Primary antibodies for the following proteins were used: PARP (9930T, Cell signalling Technology; CST, Danvers, MA, USA), cleaved PARP (9930T, CST), SHBG (sc-377032, Santa Cruz, Dallas, TX, USA), AR (5133, CST), β-actin (sc-1616, Santa Cruz), PCNA (13110, CST), EGFR (A2909, Abclonal, MA, USA), phosphor-EGFR (9789, CST), HMGB1 (CSB-PA01604A0Rb, Cusabio, Houston, TX, USA), NF-κB p65, phosphor-NF-κB p65, IκBα, and phosphor-IκBα (9936, CST).

### 4.4. Cell Culture

Human HCC SNU423 cells were used for experiment. All cell culture reagents were purchased from Welgene (Gyungsan, Korea). SNU423 cells were maintained at 37 °C in a 5% CO_2_ atmosphere in DMEM (Welgene, LM001-05) supplemented with 5% (*vol*/*vol*) fetal bovine serum, penicillin (100 U/mol), and streptomycin (100 μg/mL). Data were quantified from replicated values in which independent experiments were performed in triplicate at least.

### 4.5. Histology

Paraffin-embedded liver blocks were cut by microtome and slides were dried. After hydration steps, slides were stained by hematoxylin and eosin for H&E staining, or processed to Masson-goldner trichrome staining by using commercial kit (MGT-100T, Biognost, Zagreb, Croatia). For immunofluorescence, slides were serial-hydrated and antigen retrieved by sodium citrate buffer. After primary antibody (A5228, Sigma-Aldrich, St. Louis, MO, USA) and secondary antibody (A21202, Life Technologies, Carlsbad, CA, USA) incubation, the region of interest was observed.

### 4.6. Plasma ALT

Plasma was separated from whole blood by centrifugation and collected; 5-fold diluted plasma were used for ALT analysis. Values for diluted plasma were multiplied by 5. FUJI DRI-CHEM SLIDE (3250) and DRI-CHEM4000 (Fuji Film, Tokyo, Japan) were used for evaluation.

### 4.7. MMP9 and MMP2 Measurement

MMP2/MMP9 Gel Assay Kit (Cat#: E-118GA) purchased from Biomedical Research Service (Buffalo, NY, USA) was used for analysis. Experiments were performed according to the manufacturer’s protocol.

### 4.8. Statistical Analysis

Data are reported as mean ± SD. Differences between means were obtained by Student’s *t*-Test and the one-way ANOVA followed by a tukey’s multiple comparison test was performed using Graph Pad Software (GraphPad Inc., San Diego, CA, USA).

## Figures and Tables

**Table 1:** Primers used for real-time PCR.

Gene Name	Upper Primer (5′–3′)	Lower Primer (5′–3′)	Species
*IL-1β*	GAA ATG CCA CCT TTT GAC AGT G	CTG GAT GCT CTC ATC AGG ACA	Mouse
*Tnf*	CCT GTA GCC CAC GTC GTA G	GGG AGT AGA CAA GGT ACA ACC C	Mouse
*Ccl2*	TTA AAA ACC TGG ATC GGA ACC AA	GCA TTA GCT TCA GAT TTA CGG GT	Mouse
*Id1*	TAC GAC ATG AAC GGC TGC TA	GTG GTC CCG ACT TCA GAC TC	Mouse
*Fkbp5*	CAA AGC CTC AGA GTC GTT CC	GGA TTG ACT GCC AAC ACC TT	Mouse
*Vapa*	CAC CAG GGA TTG CTT CAA CT	AGT CGC TTG CAC TCT TCC AT	Mouse
*Ccrk*	GCT CAA AGG TGT TGC GTT TT	GTC AAC GCC CTG GTC ATA CT	Mouse
*C-Jun*	CCT TCT ACG ACG ATG CCC TC	GGT TCA AGG TCA TGC TCT GTT T	Mouse
*C-Fos*	CGG GTT TCA ACG CCG ACT A	TTG GCA CTA GAG ACG GAC AGA	Mouse
*Cyclin D*	GCG TAC CCT GAC ACC AAT CTC	CTC CTC TTC GCA CTT CTG CTC	Mouse
*Ki67*	ATC ATT GAC CGC TCC TTT AGG T	GCT CGC CTT GAT GGT TCC T	Mouse
*Ctgf*	GGG CCT CTT CTG CGA TTT C	ATC CAG GCA AGT GCA TTG GTA	Mouse
*Tgfβ1*	GAC GTC ACT GGA GTT GTA CG	GGT TCA TGT CAT GGA TGG TG	Mouse
*α-Sma*	GCT ATT CAG GCT GTG CTG TC	GGT AGT CGG TGA GAT CTC GG	Mouse
*Col1a1*	ATG TGC CAC TCT GAC TGG AA	TCC ATC GGT CAT GCT CTC TC	Mouse
*Bim*	GAC AGA ACC GCA AGG TAA TCC	ACT TGT CAC AAC TCA TGG GTG	Mouse
*Rplp0*	GCA GCA GAT CCG CAT GTC GCT CCG	GAG CTG GCA CAG TGA CCT CAC ACG G	Mouse
*Bax*	TGA AGA CAG GGG CCT TTT TG	AAT TCG CCG GAG ACA CTC	Mouse

## Data Availability

The data presented in this study are available on request from the corresponding author.

## Supplementary Materials

The following are available online at https://www.mdpi.com/article/10.3390/ijms222212557/s1.
