# Supplementary material for: Dietary Intake of 17α-Ethinylestradiol Promotes HCC Progression in Humanized Male Mice Expressing Sex Hormone-Binding Globulin
Source: Int J Mol Sci. 2021 Nov 22;22(22):12557. doi: 10.3390/ijms222212557 (PMC8620028; doi:10.3390/ijms222212557)
Supplement: Supplementary file 1 [file ijms-22-12557-s001.zip › ijms-1464754-supplementary.pdf]

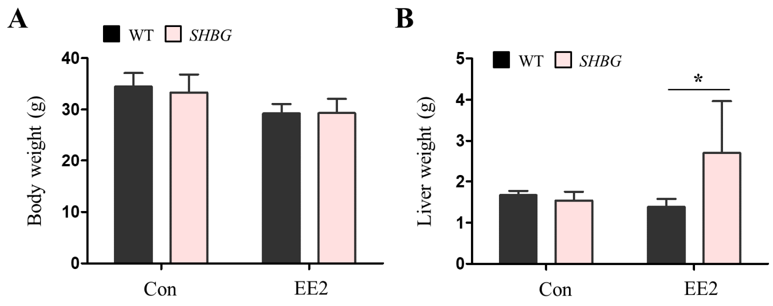

Figure S1. Body and liver weights (g) of EE2-fed WT and SHBG mice. (A) Body weight (g) of WT, SHBG, WT EE2, SHBG EE2 mice. (B) Liver weight (g) of WT, SHBG, WT EE2, SHBG EE2 mice. Numbers of mice used for experiments were: WT (4), SHBG (4), WT EE2 (8), SHBG EE2 (8). Student's t-test was used for analysis. Values represent means ± SD. *, $p<0.05$.

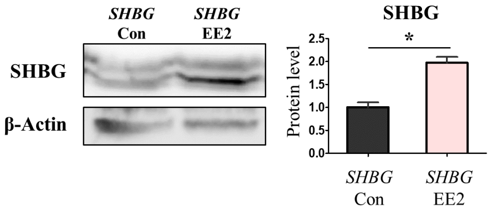

Figure S2. Hepatic SHBG was increased in *SHBG* mice fed with EE2. Western blot analysis and quantification of SHBG in livers of *SHBG* and *SHBG* EE2 mice. β-Actin was used for an internal control. Student's t-test was used for analysis. Values represent means ± SD. *, $p<0.05$. Data were quantified from replicated values in which independent experiments were performed in triplicate at least.
